# Architecture-Promoted Biomechanical Performance-Tuning of Tissue-Engineered Constructs for Biological Intervertebral Disc Replacement

**DOI:** 10.3390/ma14102692

**Published:** 2021-05-20

**Authors:** Gernot Lang, Katja Obri, Babak Saravi, Aldo R. Boccaccini, Anton Früh, Michael Seidenstücker, Bodo Kurz, Hagen Schmal, Bernd Rolauffs

**Affiliations:** 1 Department of Orthopedics and Trauma Surgery, Medical Center-Albert-Ludwigs-University of Freiburg, Faculty of Medicine, Albert-Ludwigs-University of Freiburg, Germany, Hugstetterstrasse 55, 79106 Freiburg, Germany; gernot.michael.lang@uniklinik-freiburg.de (G.L.); babak.saravi@jupiter.uni-freiburg.de (B.S.); hagen.schmal@uniklinik-freiburg.de (H.S.); 2 Institute of Biomaterials, Department of Material Science and Engineering, Friedrich-Alexander University of Erlangen-Nürnberg, Cauerstraße 6, 91058 Erlangen, Germany; katja.glier@web.de (K.O.); aldo.boccaccini@ww.uni-erlangen.de (A.R.B.); 3 AO Research Institute Davos, AO Foundation, Clavadelerstrasse 8, 7270 Davos, Switzerland; 4 G.E.R.N. Research Center for Tissue Replacement, Regeneration & Neogenesis, Department of Orthopedics and Trauma Surgery, Medical Center-Albert-Ludwigs-University of Freiburg, Faculty of Medicine, Albert-Ludwigs-University of Freiburg, Engesserstr 4, 79108 Freiburg im Breisgau, Germany; antonfrueh@aol.com (A.F.); michael.seidenstuecker@uniklinik-freiburg.de (M.S.); 5 Department of Anatomy, Christian-Albrechts-University, Otto-Hahn-Platz 8, 24118 Kiel, Germany; bkurz@anat.uni-kiel.de

**Keywords:** spine, regeneration, inflammation, intervertebral disc, tissue engineering, disc degeneration, 3R

## Abstract

Background: Biological approaches to intervertebral disc (IVD) restoration and/or regeneration have become of increasing interest. However, the IVD comprises a viscoelastic system whose biological replacement remains challenging. The present study sought to design load-sharing two-component model systems of circular, nested, concentric elements reflecting the nucleus pulposus and annulus fibrosus. Specifically, we wanted to investigate the effect of architectural design variations on (1) model system failure loads when testing the individual materials either separately or homogeneously mixed, and (2) also evaluate the potential of modulating other mechanical properties of the model systems. Methods: Two sets of softer and harder biomaterials, 0.5% and 5% agarose vs. 0.5% agarose and gelatin, were used for fabrication. Architectural design variations were realized by varying ring geometries and amounts while keeping the material composition across designs comparable. Results: Variations in the architectural design, such as lamellar width, number, and order, combined with choosing specific biomaterial properties, strongly influenced the biomechanical performance of IVD constructs. Biomechanical characterization revealed that the single most important parameter, in which the model systems vastly exceeded those of the individual materials, was failure load. The model system failure loads were 32.21- and 84.11-fold higher than those of the agarose materials and 55.03- and 2.14-fold higher than those of the agarose and gelatin materials used for system fabrication. The compressive strength, dynamic stiffness, and viscoelasticity of the model systems were always in the range of the individual materials. Conclusions: Relevant architecture-promoted biomechanical performance-tuning of tissue-engineered constructs for biological IVD replacement can be realized by slight modifications in the design of constructs while preserving the materials’ compositions. Minimal variations in the architectural design can be used to precisely control structure–function relations for IVD constructs rather than choosing different materials. These fundamental findings have important implications for efficient tissue-engineering of IVDs and other load-bearing tissues, as potential implants need to withstand high in situ loads.

## 1. Introduction

The architecture of the intervertebral disc (IVD) is complex and specific to its biomechanical demands [1,2]. Being a part of the spinal-load-bearing system [3], the IVD architecture is based on a unique multiscale extracellular matrix (ECM) structure, which allows absorption and distribution of forces that are applied to the vertebral column, essentially acting as a shock-absorber [4]. In detail, stresses, such as dynamic, static, torsional, tensile, and shear stresses, as well as their combinations, are absorbed and distributed [4] through a gelatinous, structurally and mechanically isotropic [5] central nucleus pulposus (NP) and an NP-surrounding annulus fibrosus (AF). The AF is positioned between the superior and inferior cartilaginous endplates [6,7] and has an anisotropic, inhomogeneous, multiply collagenous architecture that consists of circularly stacked lamellae of highly aligned collagen fibers [5]. Interestingly, the stacked lamellae have varying orientations that alternate above and below the transverse axis of the spine, much like angle-ply laminates that generate high shear stiffness. Moreover, the lamellae are circumferentially discontinuous and reinforced by radially outward running fibrous elements [8,9]. This complex lamellar multiscale architecture has been suggested to contain the NP under mechanical load [9]. Together, the NP and AF comprise a nonlinear viscoelastic system that provides flexibility at low loads and can simultaneously withstand higher loads while preserving structural integrity [10].

Symptomatic intervertebral disc degeneration (IDD) is among the most common causes of neck and back pain in the adult population, representing a major global epidemiological problem [11]. While first-line treatments include physical therapy and pharmacologic regimens, surgical intervention may be indicated in chronic discogenic pain or severe disease with neurologic compromise [12,13]. The current standard for surgery in IDD involves discectomy followed by the placement of an interbody graft for the fusion of the adjacent vertebrae. While well-tolerated in most patients, fusion carries a significant risk for pseudarthrosis and adjacent segment disease (ASD), which ultimately can lead to reoperations [14]. Alternatively, mechanical disc prostheses can be placed, which can preserve segmental motion and alter spine biomechanics leading to ASD. Thus, the theoretical advantage of total disc replacement over fusion surgery remains controversial [13,15]. Since disc degeneration is a multifaceted phenomenon characterized by both damages to the NP and AF, whole IVD transplantation is another treatment option that has been studied [16,17,18,19,20]. However, whether patients are treated through conservative or surgical approaches, the underlying pathophysiology leading to IDD is not addressed, resulting in a significant amount of patients suffering chronic discogenic pain [21].

To overcome the limitations of the available treatments, biological approaches to IVD restoration and/or regeneration have become of increasing interest [3,22,23]. IVD tissue engineering (TE) aims to repair the severely degenerated disc or even replace a completely degenerated segment in terminal IDD [1]. To date, much effort has been directed towards NP or AF replacements using TE technology [24,25,26]. Numerous gelatinous materials have been resourced as potential NP scaffolds, including polyurethane [24,25], alginate [27], silk–fibrin/HA composites [28], atelocollagen [29], synthetic polymers [30,31,32], and collagen II/hyaluronic/chondroitin-6-sulfate composites [33,34]. Inspired by the original AF architecture, developing AF scaffolds has focused on electrospun fibers [35], silk [28,36], and other natural fibers [26,37], and on small intestine submucosa [38]. Interestingly, in one study, an inner AF region was constructed from a concentrically wrapped biopolymer sheet seeded with chondrocytes. In contrast, an outer AF was fashioned from a demineralized bone matrix [39], illustrating a bio-inspired approach to AF TE. Another study used oriented electrospun scaffolds seeded with mesenchymal stromal cells MSCs to generate bi-lamellar constructs with opposing collagen orientations, whose tensile modulus, after 10 weeks of in vitro culture, came close to values of the native AF [40]. This study successfully mimicked angle-ply tissues, such as the AF, illustrating the value of a bio-inspired architectural design to generate both structural and functional analogs for the native tissue. In addition to the angle-ply orientation of the AF lamellae, another functionally important design characteristic of the IVD is the mechanical interplay between the NP and the AF tissues under load, as the AF receives and contains the outward-directed stress generated by the NP under mechanical strain.

Focusing on this context, the present study aimed to design simple load-sharing two-component model systems of circular, nested, concentric elements intended to broadly reflect the NP and AF manufactured from biomaterials with different mechanical stiffnesses. This was undertaken to allow studying how modifications in the architectural design affect the bulk stiffness and failure load under standardized loading conditions and, thus, to answer how architectural variations affect the overall biomechanical performance of the composite model systems while keeping the material composition across designs comparable.

The underlying hypothesis was that such architectural design variations allow (i) reaching higher model system failure loads than those of the individual materials tested either separately or homogeneously mixed, and (ii) that such variations also allow modulating other mechanical properties of the model systems.

Furthermore, caffeic acid, a novel crosslinker previously reported in food research, was used for crosslinking gelatin [41] to study its effects on gelatin mechanical properties and degradation over time and test its suitability for TE [42].

It is important to underline that the purpose of the present study was not to manufacture a highly sophisticated TE-IVD construct precisely mimicking the native structure but instead, to elucidate fundamental biomechanical interactions and, specifically, how slight modifications in the architectural design can be used to control structure–function relations for future IVD constructs.

## 2. Materials and Methods

### 2.1. Model System Architectural Design

**Figure 1:**
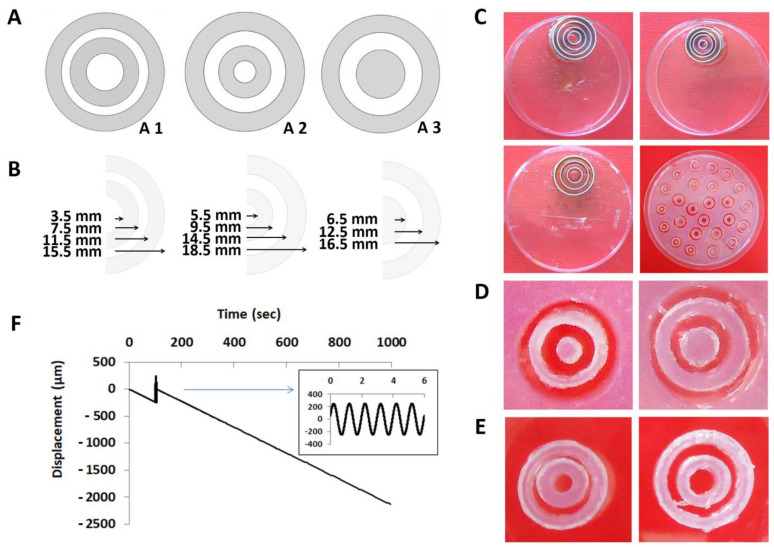
**Architectural design variations, fabrication, and mechanical characterization of load-sharing, two-component composite model systems**. (**A**) Illustration of the three architectural designs A1, A2, and A3. Dark gray illustrates elements fabricated from stiffer biomaterials, namely 5% agarose or caffeic acid-crosslinked gelatin. White illustrates elements fabricated from the softer biomaterial, 0.5% agarose. (**B**) Diameters of the central elements and inner and outer diameters of the central element-surrounding rings. The central element diameters were 3.5 mm and 5.5 mm of architectures 1 and 2 (for a soft biomaterial), the outer diameters of the first ring were 7.5 mm and 9.5 mm (for a hard biomaterial), those of the second ring were 11.5 mm and 14.5 mm (for a soft biomaterial), and those of the third ring were 15.5 mm and 18.5 mm (for a hard biomaterial). Thus, the widths of the rings intended for a hard material remained constant at 4 mm. For architecture 3, the central element diameter was 6.5 mm (for a hard biomaterial), the outer diameter of the first ring (for a soft biomaterial) was 12.5 mm and of the second ring (for a hard biomaterial) was 16.5 mm. (**C**) Three cylindrical metal punches used for model system fabrication and the 5% agarose used for punching. The punches were fabricated by gluing cylindrical metal rings in a concentric fashion onto a Petri dish. (**D**) For model system fabrication fabricated from 0.5% agarose (the soft biomaterial) and 5% agarose (the hard biomaterial), solidified 5% agarose filling the entire Petri dish with a height of 3 mm was punched, and the rings intended as space for the soft biomaterial were manually removed. The remaining space was then filled with a softer biomaterial, namely 0.5% agarose. (**E**) The rings removed from (**D**) were used as a mold for gelatin elements. After removing these rings, the empty space within the gelatin was filled with the softer biomaterial, 0.5% agarose. (**F**) illustrates the displacement over time used for mechanical characterization of the model systems, using an incubator-housed loading device IncuDyn CA2008 in displacement control and custom-made software. The sinusoidal displacement is shown enlarged. X-axis label: time (s). Y-axis label: displacement (µm).

A simple load-sharing, two-component composite model system using concentric, nested rings around a central round element, intended to broadly mimic the NP and AF, was designed. Differences in the architectural design (illustrated in Figure 1A) were realized by varying the (i) construct and ring geometries and (ii) changing the amount and order of rings while keeping the percentage of the stiff biomaterial across designs comparable.

Detailed below, architecture 1 and 2 central elements and second rings were fabricated from a soft biomaterial. In contrast, the first and third rings were fabricated from a harder biomaterial. In architecture 3, the central element and the second ring were fabricated from a harder biomaterial and the second ring from a soft biomaterial. All model systems were then covered with exactly fitting biomaterial lids as described below. These architectural design features allowed keeping the percentage of the stiff biomaterial almost constant (63.3% in architecture 1, 56.1% in architecture 2, and 58.13% in architecture 3). A homogeneous biomaterial control consisted of a homogeneous mixture of the soft and stiff biomaterials in the same ratio as in architecture 1. A so-called open control was fabricated by cutting the stiff rings of architecture 1 into pieces, which were then distributed within the soft biomaterial, leading to a structure without confinement by a stiff outer ring.

### 2.2. Model System Biomaterials

#### 2.2.1. Agarose

SeaKem^®^ LE Agarose was purchased from Cambrex Bio Science Baltimore Inc. (Baltimore, MD, USA) and dissolved at a concentration of 5% (*w*/*v*) in purified water. The solution was heated to a boiling temperature to ensure complete dissolution and then poured into Petri dishes to a final height of 3 mm (145 × 20 mm, Greiner Bio-One, Kremsmünster, Austria). The solution was allowed to cool down for 1 h to solidify and used for model system fabrication as described below and stored in phosphate-buffered saline (PBS) for 1 day before testing. Another agarose solution was produced at a concentration of 0.5% (*w*/*v*) and used as a soft biomaterial as described below.

#### 2.2.2. Gelatin

Gelatin from porcine skin (type A, Bloom strength 300, cell culture tested) was purchased (Sigma Aldrich, Darmstadt, Germany), and caffeic acid (Sigma Aldrich) was used as a novel crosslinking agent. The processing method described in [41,42] was slightly modified. 10 g gelatin was dissolved by stirring at 60 °C in 90 mL ultrapure water for 20 min. In parallel, 15 mg caffeic acid/g gelatin was dissolved in 10 mL of ultra-pure water. After 15 min, the gelatin solution pH was increased by adding 2400 µL NaOH (1 N). The solution and the dissolved caffeic acid were mixed. The pH in the resulting mixture was controlled to be pH 9, and the mixture was stirred at high speed to ensure oxygenation of the reaction solution for another 20 min at 60 °C. The mixture was then poured into Petri dishes to a final height of 3 mm and left to solidify for 72 h before the fabrication of agarose–gelatin model systems and controls. These were sterilized under UV light for 15 min and stored in PBS with added amphotericin B (1.06%) and penicillin–streptomycin (1.77%) (both Life Technologies, Carlsbad, CA, USA) for 1 day before testing.

### 2.3. Model System Fabrication

To broadly mimic NP and AF differences in stiffness, we used biomaterials with different stiffnesses, 0.5% agarose vs. 5% agarose for the first set of model systems, and 0.5% agarose vs. gelatin for the second set of the model system. Thus, we manufactured two composite model systems, each with three different architectures and two controls as detailed above. The sample size was set to *n* = 6 each. First, punches were fabricated by gluing custom-made cylindrical rings on the lid of a Petri dish. These cylindrical rings were made by the Department of Chemistry, University of Tübingen, Germany, from aluminum, having a height of 5 mm, a thickness of 0.5 mm, and variable diameters of 1 to 20 mm. The punches shown in Figure 1C were used to produce 5% agarose molds.

For model system fabrication made of 0.5% agarose (the soft biomaterial) and 5% agarose (the hard biomaterial), solidified 5% agarose filling the entire Petri dish with a height of 3 mm was punched. The rings intended as space for the soft biomaterial were manually removed (an example is given in Figure 1D). The removed rings intended as hard biomaterial (an example is given in Figure 1E) were superglued (Pattex Sekundenkleber ultra gel, Henkel, Germany) to a 5% agarose bottom of 1 mm height in a concentric, nested assembly. The empty spaces intended for the soft biomaterial were then filled with 0.5% agarose, and a 5% agarose lid of 1 mm height was superglued. The model bottom and lid were punched from 5% agarose that had solidified in another Petri dish.

For model system fabrication made of 0.5% agarose (the soft biomaterial) and gelatin (the hard biomaterial), the rings that were removed during the agarose model system fabrication were reused to form the mold for hard gelatin rings. The empty spaces of the mold intended for the hard biomaterial were then filled with gelatin. Subsequently, the mold rings were removed, the gelatin rings were superglued to a gelatin bottom of 1 mm height, and the remaining empty spaces intended for the soft biomaterial were filled with 0.5% agarose. Finally, a gelatin lid of 1 mm height was superglued. In another Petri dish, gelatin having solidified was used for punching the model bottom and lid.

### 2.4. Model System Mechanical Characterization

All model systems were tested in unconfined compression in a displacement-controlled, incubator-housed device (IncuDyn CA 2008, custom-designed by the Massachusetts Institute of Technology Center for Biomedical Engineering, Cambridge, MA, USA) controlled by the custom-written software DACQSP (version 9.012D build 162, custom-designed by the Massachusetts Institute of Technology Center for Biomedical Engineering, Cambridge, MA, USA) [43] and with an upper non-porous platen that was larger than the individual model system to characterize bulk behavior (Supplementary Figure S1). For all mechanical tests, the testing protocol begun with a linear compression ramp of 5% strain in 100 s, followed by a sinusoidal compression with an amplitude of 3% and a frequency of 1 Hz for 6 s, and, finally, a final linear compression ramp to 50% compression in 900 s, amounting to a total testing time of 1006 s. An illustration of the displacements that were applied over the entire characterization time is given in Figure 1F. No relaxation time was allocated. The resulting data were used to calculate the compressive strength, the dynamic stiffness, and the load at failure. The apparent compressive strength was calculated using the slope of a linear fit to the stress–strain curve that resulted from the first compression ramp. The dynamic stiffness was calculated from the sinusoidal compression step as described in [44,45]. The viscoelastic properties were determined by calculating the phase shift between stress and strain. The load at failure was defined as the load measured when the first change in the slope of the stress–strain curve occurred, indicating structural failure [44].

### 2.5. Gelatin Degradation Behavior

To determine the degradation behavior of crosslinked gelatin, homogeneous constructs with 20 mm diameter and 5 mm height fabricated from gelatin crosslinked with caffeic acid were tested for functional degradation by mechanically characterizing the systems at room temperature and at 37 °C at days 1, 3, 7, and 14. To determine weight loss during degradation, the samples were dried using a SpeedVac concentrator (SpeedVac SC 200, Savant Instruments Inc., Farmingdale, NY, USA). After drying, the mass of the degraded samples was measured using an electronic balance (Sartorius handy H51, Sartorius Weighing Technology GmbH, Göttingen, Germany).

### 2.6. Cell Isolation and Culture

Articular cartilage was obtained with institutional approval and informed consent (713/2012BO2) from the knee joints of six patients diagnosed with osteoarthritis during joint replacement. Human chondrocytes and chondrons, which are chondrocytes and their native surrounding pericellular matrix (PCM), were isolated from human condylar articular cartilage. The resulting chondrocytes were used for cytotoxicity tests. The resulting chondrons were used for seeding a model system (see below). As described in [46], using 0.8 mg/mL Dispase-II (Roche, Mannheim, Germany) and 0.2 mg/mL collagenase-XI (Sigma-Aldrich, Darmstadt, Germany), OA chondrocytes were isolated overnight at 37 °C. For OA chondron isolation, 1.8 mg/mL Dispase-II (Roche, Mannheim, Germany) and 2 mg/mL collagenase-P (Roche, Mannheim, Germany) were used for 5 h at 37 °C. The digest was filtered with a 100 µm cell strainer (Fisher Scientific) and transferred to a cell culture flask (CELLSTAR Filter cap cell culture flasks, 75 cm^2^, Greiner Bio-One, Kremsmünster, Austria) for chondrocyte culture at 37 °C and 5% CO_2_ in 20 mL standard medium consisting of 1:1 GlutaMAX with F12 and GlutaMAX with high glucose DMEM (Gibco, Gaithersburg, MD, USA), 10% FBS superior, 2% pen–strep (Gibco), 1% fungicide (Biochrom, Berlin, Germany), and 0.1% L-ascorbic acid (Sigma-Aldrich). The medium was changed every second day. Chondrons were kept in a highly concentrated suspension in the medium at 4 °C overnight before seeding (see Supplementary Table S3 for the composition of the chondrocyte cultivation medium and Supplementary Table S4 for the composition of the digestion solution for chondron isolation).

### 2.7. Caffeic Acid-Crosslinked Gelatin Cytotoxicity

To test the biocompatibility of caffeic acid as a crosslinker, homogeneous constructs with 20 mm diameter and 5 mm height fabricated from gelatin crosslinked with caffeic acid were stored in PBS for 5 days with a change of PBS every second day to allow pH equilibration. After 5 days, the PBS was replaced with the standard medium as described, and the test systems were incubated in medium for 3 days at 37 °C, 5% CO_2_. Human chondrocytes isolated as described above were detached from their flask between days 5 and 7 using the trypsin-EDTA solution and seeded in a 6-well plate at a density of 5000 cells/well. After 2 days, the crosslinked gelatin constructs were placed into the wells and on top of the chondrocytes. Cell survival was assessed on day 11 (see below: Live-Dead Imaging).

### 2.8. Model System Architecture 1 from 5% Agarose and a Chondron-Seeded Dextran Hydrogel

To test a model system seeded with human cells, architecture 1 was generated from 5% agarose as hard biomaterial and a chondron-seeded dextran hydrogel (3D-Life-Dextran-PEG, Cellendes, Reutlingen, Germany) as a soft biomaterial. The dextran hydrogel consists of a maleimide-functionalized polymer, here dextran, and a thiol-functionalized crosslinker, here a thiol-functionalized polyethylene glycol (see Supplementary Table S1 for the composition of the 3D-life hydrogel). Dextran hydrogels were prepared according to the manufacturer’s protocol and according to our previous publication [46]. In detail, 1.5 µL distilled water, 2.5 µL 10× Buffer (pH 5.5), 5 µL maleimide–dextran (30 mM), 10 µL RGD-peptide (3 mM) were mixed and kept at room temperature for 10 min. 5 µL chondron suspension in PBS at a concentration of 16 × 10^6^ cells/mL and then 6 µL PEG-link (20 mM) were added to the hydrogel (see Supplementary Table S2 for the reagents for the 3D-life hydrogel).

Architecture 1 was pre-fabricated from 5% agarose as described above, except fibrin glue (Tissucol Duo S 2 mL Immuno, Baxter, Deerfield, IL, USA) was used instead of superglue and filled with the chondron-loaded dextran hydrogel. Since the gelation occurred quickly, the pre-portioned PEG-link amounts were mixed with accordingly pre-portioned reaction solutions separately for each test system ring space in a pipette tip before introducing the hydrogel into the system.

The top of the fabricated model system was kept open to ensure sufficient gas exchange, and the system was cultured for 7 days using the above-described medium changed twice. Then, a pre-compression of 5% was applied, followed by a sinusoidal compression with an amplitude of 5% and a frequency of 1 Hz for 5 min. This protocol produced a total strain ranging from 0 to 10% to represent strains from flexion to extension of the spine in the IVD [47]. Non-loaded test systems served as controls. All constructs were then incubated for another day. Cell survival was tested on day 8 in all scaffolds using the cell viability imaging kit (Roche, Mannheim, Germany).

### 2.9. Live-Dead Imaging

Fluorescence microscopy was used combined with the cell viability imaging kit (Roche, Switzerland) to assess cell viability. Nuclei were stained by Hoechst 33342 (Thermo Fisher Scientific, Dreieich, Germany), living cells by calcein and dead cells by propidium iodide (PI) according to the manufacturer’s protocol. Mosaic images of the stained cells were generated using an Axio Observer Z1 fluorescence microscope with a motorized stage (Zeiss, Oberkochen, Germany) and the corresponding software (AxioVision 4.8.2, Carl Zeiss, Gottingen, Germany). Images were analyzed using ImageJ [48]. Cell survival was calculated according to Equation (1):

(1) $$S=1-\frac{n\;\left(dead\;cells\right)}{n\;\left(live\;cells\right)}$$

### 2.10. Statistical Analysis

All data are presented as mean ± standard error of the mean (SEM) in the text of the results section or as box plots in the figures. Box plots were plotted using SigmaStat (Systat Software, Version 12.5, San Jose, CA, USA) and give the median and the 25th and 75th percentiles. In contrast, the whiskers give the 10th and 90th percentiles. All mechanical data are shown inverted due to convention. Calculations were performed with Microsoft Excel Version 14.0.7258.5000 (Microsoft, Redmond, WA, USA). All statistical tests were performed using SigmaStat 12.5 (Systat Software, Version 12.5, San Jose, CA, USA). Two groups were compared using a Mann–Whitney rank-sum test when data failed the Shapiro–Wilk normality test or the equal variance test. A *t*-test was used when data were normally distributed and had equal variance. More than two groups were compared using the Kruskal–Wallis one-way analysis of variance on ranks test (ANOVA on ranks) when data were not normally distributed (Shapiro–Wilk test) and an analysis of variance test (ANOVA) when data were normally distributed. As a pairwise multiple comparison procedure, the Student–Newman–Keuls Method was used for ANOVA. Dunn’s method was used as a pairwise multiple comparison procedure for ANOVA on ranks when the sample size was unequal across groups. The Student–Newman–Keuls method was used when the sample size was equal across groups. The given *p*-values refer to the pairwise multiple comparison procedures. Differences were considered significant at *p* < 0.05. The term “best-performing” architecture was defined as the architecture with the highest average value in either failure load, compressive strength, dynamic stiffness or phase shift.

## 3. Results

All architectures and controls were successfully fabricated and characterized as described above.

### 3.1. Load at Failure

**Figure 2:**
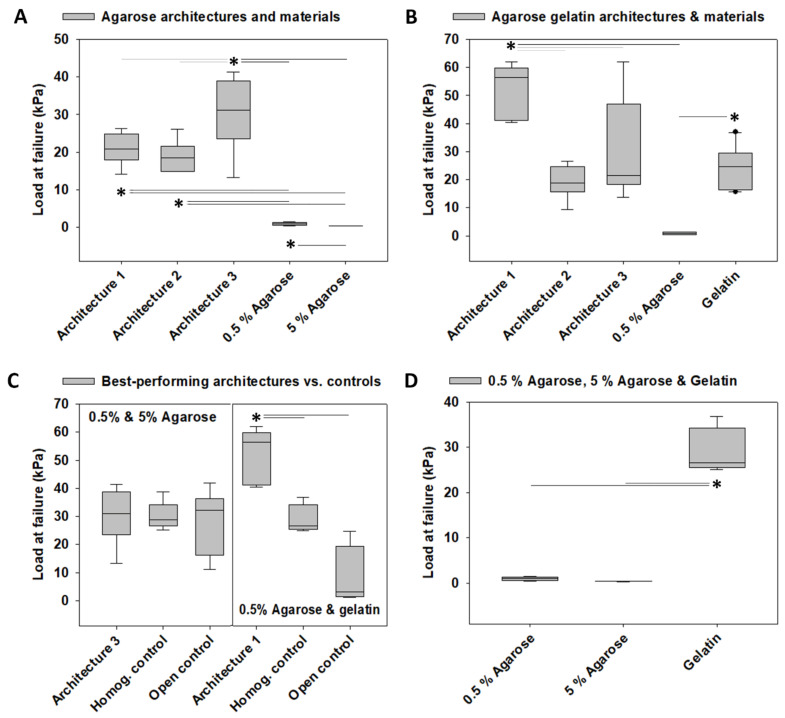
**Mechanical characterization of the failure loads of the tested model systems, controls, and materials used for fabrication.** (**A**) Failure loads of the architectures 1, 2, and 3 of the 0.5% and 5% agarose model system and of the biomaterials used for fabrication. (**B**) Failure loads of the architectures 1, 2, and 3 of the 0.5% agarose gelatin model system and of the biomaterials used for fabrication. (**C**) Failure loads of the best-performing architecture of both agarose model systems and agarose gelatin model systems determined in (**A**,**B**) as well as their associated homogeneously mixed controls and open controls. Those were fabricated by cutting the stiff rings of architecture 1 into pieces and distributing them within the softer biomaterial, leading to an unconfined situation. (**D**) Failure loads of all individual materials used for model system fabrication. The box plots give the median and the 25th and 75th percentiles, the whiskers give the 10th and 90th percentiles, and the round symbols, if present, indicate outliers. The dark gray lines and the asterisks (*) indicate differences at *p* < 0.05 identified in ANOVA or ANOVA on ranks and pairwise multiple comparison procedures, such as the Newman–Keuls or Dunn’s method. Light gray lines and the asterisk (*) indicate differences at *p* < 0.05 that were identified with the same statistical tests, in which only architectures 1, 2, and 3 were compared. Number of 0.5% and 5% agarose homogeneous controls: *n* = 5, number of all other 0.5% and 5% agarose samples: *n* = 6, number of gelatin material samples: *n* = 10, number of all other 0.5% agarose and gelatin samples: *n* = 6.

The model system architectures fabricated from 0.5% and 5% agarose exhibited failure loads of 20.93 ± 1.58 kPa (architecture 1), 18.84 ± 1.58 kPa (architecture 2), and 30.28 ± 3.68 kPa (architecture 3) that were higher than from those of the individual materials, of which the architectures consisted (0.5% agarose: 0.94 ± 0.15 kPa, 5% agarose: 0.36 ± 0.01 kPa, *p* < 0.05). The failure loads of those materials, 0.5% and 5% agarose were also different (*p* < 0.05). Moreover, the failure load of architecture 3, the architecture with the highest failure load, was 32.21-fold higher than 0.5% agarose and 84.11-fold higher than 5% agarose. When we compared only the architectures 1, 2, and 3 in a separate test, the architectures were also different from each other (*p* < 0.05), revealing a modulation of failure load by architecturally varying construct design parameters without altering construct biomaterial composition. The results of all pairwise multiple comparison procedure tests are illustrated in Figure 2A.

The model system architecture 1 fabricated from 0.5%, and gelatin exhibited a failure load of 51.73 ± 3.94 kPa, which was higher than the failure load of 0.5% agarose (0.94 ± 0.15 kPa, *p* < 0.05). The two materials of which this architecture consisted were also different from each other (gelatin: 24.18 ± 0.15 kPa, *p* < 0.05). Comparing architectures 1, 2, and 3 revealed that the failure load of architecture 1 was different from that of architectures 2 (19.26 ± 2.25 kPa) and 3 (30.07 ± 6.82 kPa, *p* < 0.05). This indicated modulation of failure load by architecturally varying construct design parameters without altering construct biomaterial composition. Interestingly, the failure load of architecture 1 was 55.03-fold higher than 0.5% agarose and 2.14-fold higher than gelatin. The results of all pairwise multiple comparison procedure tests are illustrated in Figure 2B.

We then tested the best-performing architectures against a homogeneous control fabricated as a homogeneous mixture of the soft and stiff biomaterials in the same ratio as in architecture 1 and against an open control fabricated from cutting the stiff rings of architecture 1 into pieces and distributing them within the soft biomaterial. In terms of the highest failure load, the best-performing architecture fabricated from 0.5% and 5% agarose was architecture 3. However, its failure load was not different from those of the homogeneous and open controls. This was in contrast to architecture 1, the best-performing architecture of the 0.5% agarose and gelatin architectures, whose failure load was higher than that of both the homogeneous and open controls (*p* < 0.05). Moreover, the architecture 1 failure load was 55.03-fold higher than the failure load of 0.5% agarose and 2.14-fold higher than the failure load of gelatin, the biomaterial that was chosen as harder material. The results of all pairwise multiple comparison procedure tests are illustrated in Figure 2C.

The failure loads of 0.5% agarose and 5% agarose were different from each other when we tested both materials and the three resulting architectures, as illustrated in Figure 2A (*p* < 0.05). When comparing all three materials, 0.5% agarose, 5% agarose, and gelatin, in a separate test, the gelatin failure load was different from the other two materials (*p* < 0.05). The results of these pairwise multiple comparison procedure tests are illustrated in Figure 2D.

Next, we defined the architecture with the highest average value as “best-performing” architecture. We compared the best-performing architectures of the agarose vs. agarose gelatin sets. Importantly, this test revealed that the failure load of architecture 1 fabricated from 0.5% agarose and gelatin was 1.71-fold higher than that of architecture 3 fabricated from 0.5% and 5% agarose (*p* < 0.01). This was likely based on the differences in the failure loads that we found between the two materials used as harder biomaterials in the two architecture sets, 5% agarose and gelatin (*p* = 0.001), as gelatin exhibited a 67.23-fold higher failure load than 5% agarose.

Even more interesting was the finding that the 0.5% and 5% agarose architecture 3 exhibited a 32.21-fold higher failure load than 0.5% agarose (*p* < 0.05) and an 84.11-fold higher failure load than 5% agarose (*p* < 0.05). Similarly, the 0.5% agarose and gelatin architecture 1 exhibited a 55.03-fold higher failure load than 0.5% agarose (*p* < 0.05) and a 2.14-fold higher failure load than gelatin, but this latter comparison did not reach significant levels. Overall, these data indicated that incorporating architectural design features into the construct led to improved failure loads that vastly exceeded those of the individual materials used for fabrication.

Finally, we compared the open controls to their respective architecture 1 because the open control was fabricated by cutting the stiff rings of architecture 1 into pieces, which were then distributed within the soft biomaterial. This led to a situation where architecture 1 materials and component dimensions were kept constant but with a spatially different distribution, namely with vs. without an outer ring. The failure load of architecture 1 vs. open control was different for the agarose gelatin set (*p* < 0.05) but comparable for the agarose set, indicating a pronounced material effect in the performance of the two sets because, in these two comparisons, all factors except the choice of materials were kept constant. Moreover, the failure loads of the two open controls were different (*p* < 0.05) and also of architecture 1 made from agarose vs. 0.5% agarose and gelatin, indicating together a failure behavior-modulating effect of the choice of material, as all other factors were kept constant. However, the failure loads of the two homogeneous controls were not different, which suggested that the effects of architectural design were more pronounced than the effects of the choice of materials on failure behavior.

### 3.2. Compressive Strength

**Figure 3:**
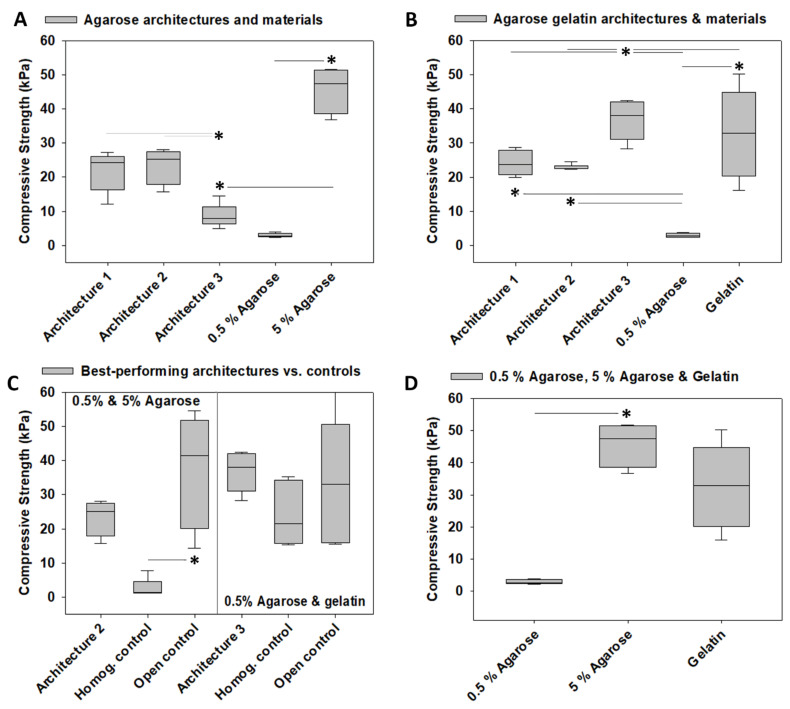
**Mechanical characterization of the compressive strengths of the tested model systems, controls, and materials used for fabrication**. (**A**) Compressive strengths of the architectures 1, 2, and 3 of the 0.5% and 5% agarose model system and of the biomaterials used for fabrication. (**B**) Compressive strengths of the architectures 1, 2, and 3 of the 0.5% agarose gelatin model system and of the biomaterials used for fabrication. (**C**) Compressive strengths of the best-performing architecture of both agarose model systems and agarose gelatin model systems determined in (A) and (B) as well as their associated homogeneously mixed controls and open controls. Those were fabricated by cutting the stiff rings of architecture 1 into pieces and distributing them within the softer biomaterial, leading to an unconfined situation. (D) Compressive strengths of all individual materials used for model system fabrication. The asterisk (*) indicates a significant difference (*p* < 0.05). The box plots give the median and the 25th and 75th percentiles. The whiskers give the 10th and 90th percentiles, and the round symbols indicate outliers if present. The dark gray lines and the asterisks (*) indicate differences at *p* < 0.05 identified in ANOVA or ANOVA on ranks and pairwise multiple comparison procedures, such as the Newman–Keuls or Dunn’s method. Light gray lines and asterisks (*) indicate differences at *p* < 0.05 that were identified with the same statistical tests. Only architectures 1, 2, and 3 were compared. Number of 0.5% and 5% agarose architecture 1 model systems: *n* = 5, number of 0.5% and 5% agarose architecture 2 model systems: *n* = 4, number of 0.5% and 5% agarose architecture 3 model systems: *n* = 6, number of 0.5% and 5% agarose homogeneous controls: *n* = 5, number of 0.5% and 5% agarose open controls: *n* = 5, number of 0.5% agarose material samples: *n* = 5, number of 5% agarose material samples: *n* = 4, number of all 0.5% agarose and gelatin samples: *n* = 6 each.

The model system architectures fabricated from 0.5% and 5% agarose exhibited a compressive strength of 21.81 ± 2.38 kPa (architecture 1), 23.52 ± 2.33 kPa (architecture 2), and 8.67 ± 1.25 kPa (architecture 3), whereas 2.93 ± 0.23 kPa was the compressive strength of 0.5% agarose and 45.86 ± 3.01 kPa was that of 5% agarose. Statistically comparing the compressive strengths of these biomaterials indicated differences between the two agarose concentrations and revealed that the compressive strength of architecture 3 was lower than that of 5% agarose *p* < 0.05). However, the compressive strengths of the three agarose architectures were largely in between those calculated for the two materials, 0.5% and 5% agarose. Comparing the three architectures revealed that architecture 3 compressive strength was lower than those of the other two architectures (*p* < 0.05), again indicating a modulation of compressive strength by architecturally varying construct design parameters without altering construct biomaterial composition. The results of the pairwise multiple comparison tests are illustrated in Figure 3A. When calculating the compressive strength of the 0.5% and 5% agarose model systems, we noted that one sample of architecture 1 and one sample of architecture 2 exhibited a sudden change in slope followed by another linear increase of stress with strain in their stress–strain curves over time. We interpreted this as imprecise manufacturing (data not shown) and excluded these samples from the analyses. This problem was not observed in any of the 0.5% agarose and gelatin architectures.

In principle, the model system architectures fabricated from 0.5% and gelatin exhibited a compressive strength profile comparable to that of the agarose architectures. Importantly, the architecture 3 compressive strength (36.80 ± 2.14 kPa) was higher than that of architecture 1 (24.14 ± 1.56 kPa) and architecture 2 (22.85 ± 0.31 kPa, *p* < 0.05), indicating a modulation of failure load by architecturally varying construct design parameters without altering construct biomaterial composition. The compressive strengths of the three architectures were largely in between those calculated for the two materials used for architecture fabrication, 0.5% agarose and gelatin. In detail, the compressive strength of architectures 2 and 3 was higher than that of 0.5% agarose (*p* < 0.05). In contrast, the compressive strength of architecture 3 was different from that of both 0.5% agarose and gelatin (*p* < 0.05). The results of the pairwise multiple comparison tests are illustrated in Figure 3B.

**Figure 4:**
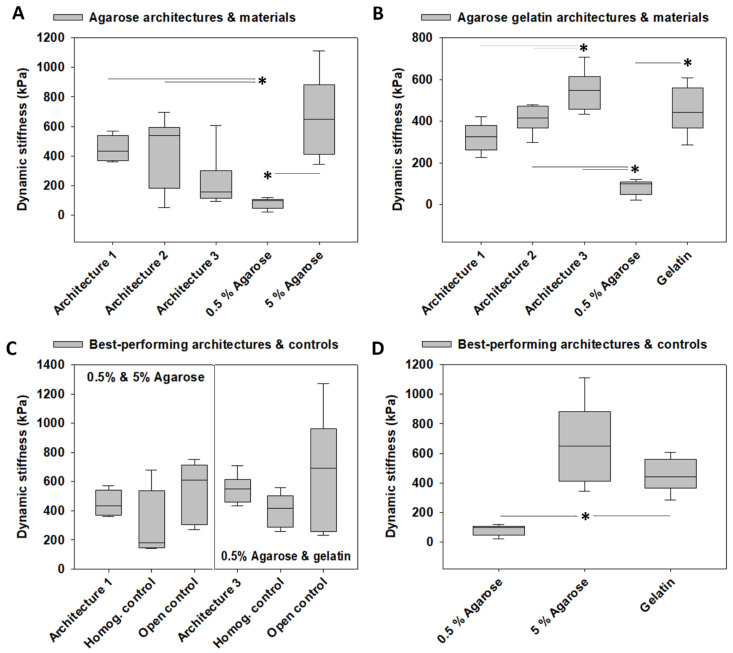
**Mechanical characterization of the dynamic stiffness of the tested model systems, controls, and materials used for fabrication**. (**A**) Dynamic stiffness of the architectures 1, 2, and 3 of the 0.5% and 5% agarose model system and of the biomaterials used for fabrication. (**B**) Dynamic stiffness of the architectures 1, 2, and 3 of the 0.5% agarose gelatin model system and of the biomaterials used for fabrication. (**C**) Dynamic stiffness of the best-performing architecture of both agarose model systems and agarose gelatin model systems determined in (**A**,**B**) as well as their associated homogeneously mixed controls and open controls. Those were fabricated by cutting the stiff rings of architecture 1 into pieces and distributing them within the softer biomaterial, leading to an unconfined situation. (**D**) Dynamic stiffness of all individual materials used for model system fabrication. The box plots give the median and the 25th and 75th percentiles, the whiskers give the 10th and 90th percentiles, and the round symbols if present indicate outliers. The dark gray lines and the asterisks (*) indicate differences at *p* < 0.05 identified in ANOVA or ANOVA on ranks and pairwise multiple comparison procedures, such as the Newman–Keuls or Dunn’s method. Light gray lines and the asterisk (*) indicate differences at *p* < 0.05 that were identified with the same statistical tests, in which only architectures 1, 2, and 3 were compared. Number of 0.5% and 5% agarose architecture 1 model systems: *n* = 6, number of 0.5% and 5% agarose architecture 2 model systems: *n* = 7, number of 0.5% and 5% agarose architecture 3 model systems: *n* = 6, number of 0.5% and 5% agarose homogeneous controls: *n* = 5, number of 0.5% and 5% agarose open controls: *n* = 6, number of 0.5% agarose material samples: *n* = 8, number of 5% agarose material samples: *n* = 6, number of 0.5% agarose and gelatin architecture 1 model systems: *n* = 5, number of 0.5% agarose and gelatin architecture 2 model systems: *n* = 6, number of 0.5% agarose and gelatin architecture 3 model systems: *n* = 6, number of 0.5% agarose and gelatin homogeneous controls: *n* = 6, number of 0.5% agarose and gelatin open controls: *n* = 6, number of 0.5% agarose material samples: *n* = 8, number of gelatin material samples: *n* = 7.

We then defined the architecture with the highest average value as “best-performing” architecture. Testing the best-performing architecture against the homogeneous and open controls in terms of compressive strength, the best-performing architecture fabricated from 0.5% and 5% agarose was architecture 2. However, its compressive strength was in between the values for the two controls and not different from them, whereas the 0.5% and 5% agarose homogeneous and open controls were different from each other (*p* < 0.05). Comparable results were found for the 0.5% agarose and gelatin architecture 3, as its compressive strength was in between the values of the homogeneous and open controls, whose compressive strengths were, however, comparable, presumably because the compressive strength of gelatin was in between the values for 0.5% and 5% agarose. The results of the pairwise multiple comparison tests are illustrated in Figure 3C and Figure 4D.

Finally, we compared the best-performing 0.5% and 5% agarose architecture 2 against the best-performing 0.5% agarose and gelatin architecture 3, which revealed that the compressive strength of the gelatin architecture 3 was 1.56-fold higher than that of the agarose architecture 2 (*p* < 0.05). Interestingly, this was not based on individual materials because gelatin did not exhibit higher compressive strength than 5% agarose. Instead, the compressive strength of gelatin was calculated to be 0.72-fold of that of 5% agarose, but this difference did not reach significance.

In contrast to the findings on failure load, in which the best-performing architectures outperformed the individual materials used for fabrication, the best-performing architectures in terms of compressive strength did not exceed the individual materials, indicating that incorporating architectural design features into the construct did not lead to improved compressive strengths and, thus, those of the individual materials used for fabrication were not exceeded.

### 3.3. Dynamic Stiffness

The model system architectures fabricated from 0.5% and 5% agarose exhibited a dynamic stiffness of 450.16 ± 33.12 kPa (architecture 1), 413.09 ± 96.09 kPa (architecture 2), and 222.43 ± 71.90 kPa (architecture 3). The dynamic stiffness of 0.5% agarose was 83.52 ± 11.25 kPa and of 5% agarose 666.36 ± 104.88 kPa. Comparing the dynamic stiffness of these biomaterials indicated differences between the two agarose concentrations. This demonstrated that the dynamic stiffness of architectures 1 and 2 was higher than that of 0.5% agarose *p* < 0.05) and comparable to that of 5% agarose. Overall, the dynamic stiffness of the three agarose architectures was largely in between those calculated for 0.5% and 5% agarose and not different from each other. Comparing the three agarose architectures did not reveal any differences in the dynamic stiffness of the three architectures, indicating that architecturally varying construct design parameters with constant construct biomaterial composition did not have effects on the dynamic stiffness of agarose constructs. The results of the pairwise comparison tests are illustrated in Figure 4A.

The model system architectures fabricated from 0.5% and gelatin exhibited a comparable profile to that of the agarose architectures, with two exceptions. First, the architecture 3 dynamic stiffness (547.87 ± 36.07 kPa) was higher than that of architecture 1 (321.00 ± 59.28 kPa) and architecture 2 (411.27 ± 25.38 kPa, *p* < 0.05), indicating a modulation of dynamic stiffness by architecturally varying construct design parameters of agarose gelatin constructs. Second, the dynamic stiffness of 0.5% agarose and gelatin architectures increased across architectures 1, 2 and 3. In contrast, the dynamic stiffness of 0.5% and 5% agarose architectures exhibited a tendency to decrease across architectures 1, 2, and 3, but these changes did not reach significance. Moreover, the dynamic stiffness of architectures 2 and 3 was higher than that of 0.5% agarose (*p* < 0.05). In contrast, the dynamic stiffness of all architectures was comparable to that of gelatin. Comparing the dynamic stiffness of the two biomaterials used for fabrication indicated differences between them, as gelatin exhibited a 4.49-fold higher dynamic stiffness than 0.5% agarose (*p* < 0.05). The results of the pairwise multiple comparison tests are illustrated in Figure 4B.

We then defined the architecture with the highest average value as “best-performing” architecture. Testing the best-performing architecture against the homogeneous and open controls in terms of dynamic stiffness revealed that neither architecture 1 fabricated from 0.5% and 5% agarose nor architecture 3 was different from the two controls, as the dynamic stiffness of both architectures was in between the values of the two associated controls (Figure 4C). Notably, the homogeneous and open controls were not different from each other.

Finally, we compared the best-performing 0.5% and 5% agarose architecture 1 against the best-performing 0.5% agarose and gelatin architecture 3, which, however, revealed no differences in the dynamic stiffness. Interestingly, the materials used for fabricating these architectures did exhibit differences in their dynamic stiffness when comparing 5% agarose with 0.5% agarose and when comparing 5% agarose with gelatin (*p* < 0.05). Thus, in terms of dynamic stiffness, the best-performing architectures did not exceed the individual materials, indicating that incorporating architectural design features into the construct did not lead to improved dynamic stiffnesses. The results of the pairwise multiple comparison tests are illustrated in Figure 4D.

### 3.4. Viscoelastic Properties

The viscoelastic properties were assessed by calculating the phase shift *δ* between stress and strain under dynamic sinusoidal load. The model system architectures fabricated from 0.5% and 5% agarose exhibited a phase shift of 0.07 ± 0.01 kPa (architecture 1), 0.13 ± 0.03 kPa (architecture 2), and 0.14 ± 0.04 kPa (architecture 3). The phase shift of 0.5% agarose was 0.25 ± 0.04 kPa and of 5% agarose was 0.08 ± 0.02 kPa.

**Figure 5:**
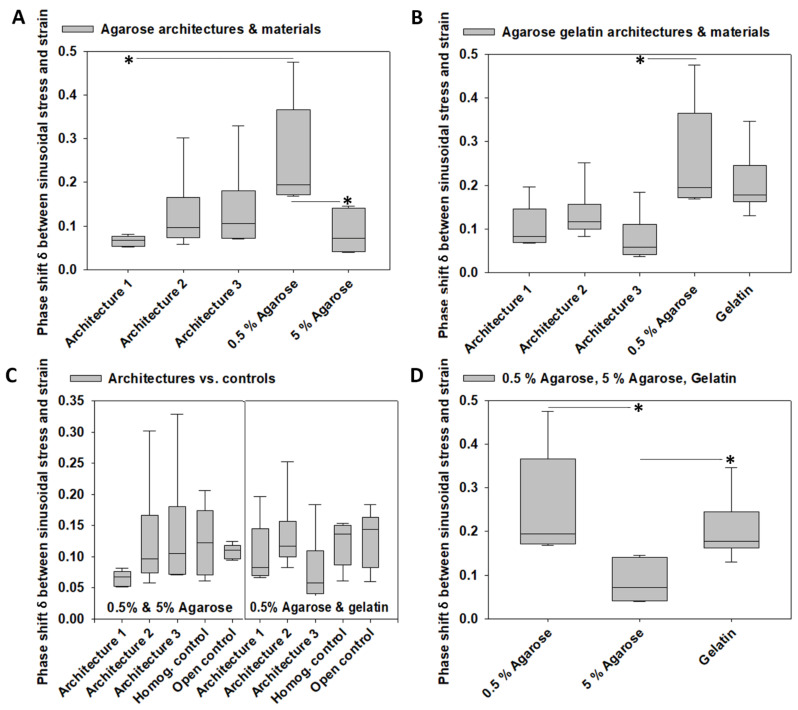
**Mechanical characterization of the phase shift as an indicator of the viscoelasticity of the tested model systems, controls, and materials used for fabrication**. (**A**) Phase shift of the architectures 1, 2, and 3 of the 0.5% and 5% agarose model system and of the biomaterials used for fabrication. (**B**) Phase shift of the architectures 1, 2, and 3 of the 0.5% agarose gelatin model system and of the biomaterials used for fabrication. (**C**) Phase shift of all architectures of both agarose model systems and agarose gelatin model systems determined in (**A**,**B**) as well as their associated homogeneously mixed controls and open controls. Those were fabricated by cutting the stiff rings of architecture 1 into pieces and distributing them within the softer biomaterial, leading to an unconfined situation. (**D**) Phase shift of all individual materials used for model system fabrication. The box plots give the median and the 25th and 75th percentiles. The whiskers give the 10th and 90th percentiles, and the round symbols indicate outliers if present. The dark gray lines and the asterisks (*) indicate differences at *p* < 0.05 identified in ANOVA or ANOVA on ranks and pairwise multiple comparison procedures, such as the Newman–Keuls or Dunn’s method. Number of 0.5% and 5% agarose architecture 1, 2, and 3 model systems: *n* = 6 each, number of 0.5% and 5% agarose homogeneous controls: *n* = 5, number of 0.5% and 5% agarose open controls, 0.5% and 5% agarose material samples: *n* = 6 each, number of 0.5% agarose and gelatin architecture 1 model systems: *n* = 5, number of 0.5% agarose and gelatin architecture 2 and 3 model systems: *n* = 6 each, number of 0.5% agarose and gelatin homogeneous and open controls: *n* = 6 each, number of 0.5% agarose material samples: *n* = 6, number of gelatin material samples: *n* = 10.

The phase shift was different between the two agarose concentrations (*p* < 0.05) and between 0.5% agarose and architecture 1 (*p* < 0.05), which exhibited the lowest value of all architectures and biomaterials used for fabrication. The phase shift of the three agarose architectures was not different and largely between the values of the 0.5% and 5% agarose, indicating that architecturally varying construct design parameters with constant construct biomaterial composition did not have effects on the phase shift of agarose constructs. The results of the pairwise multiple comparison tests are illustrated in Figure 5A.

We did not find any differences in the phase shift between the three agarose gelatin architectures and the two biomaterials used for fabrication, 0.5% and gelatin, except that architecture 3 had a smaller phase shift than 0.5% agarose (*p* < 0.05). The results of the pairwise multiple comparison tests are illustrated in Figure 5B.

Because the phase shift of the three architectures of the agarose or the agarose gelatin sets were all in the same range, no best-performing architecture was selected. Thus, we compared all architectures to their associated homogeneous and open controls, which, however, revealed no differences and illustrated that the phase shift of all architectures was largely in between the values of their two associated controls (Figure 5C), indicating that architecturally varying construct design parameters with constant construct biomaterial composition did not have effects on the phase shift of the agarose and the agarose gelatin constructs. Moreover, the homogeneous and open controls were not different from each other. The materials used for fabricating the architectures exhibited differences in their phase shift when comparing 0.5% agarose with 5% agarose and when comparing 5% agarose with gelatin (*p* < 0.05). The results of the pairwise multiple comparison tests are illustrated in Figure 5D.

### 3.5. Mechanical Characterization of Gelatin over Time at Room Temperature vs. 37 °C

**Figure 6:**
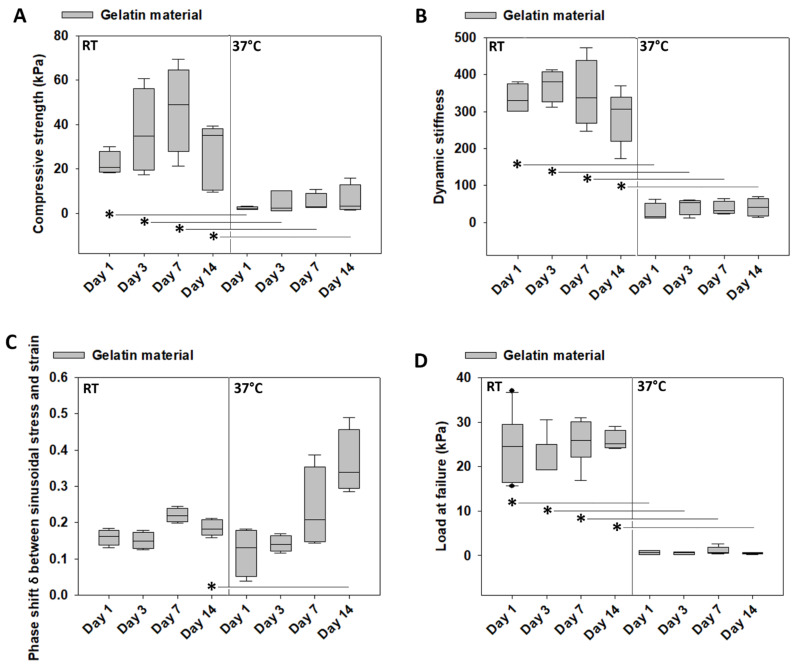
**Mechanical characterization of homogeneous constructs with 20 mm diameter and 5 mm height fabricated from gelatin crosslinked with caffeic acid to determine the degradation behavior at room temperature (RT) and at 37 °C over time**. (**A**) Compressive strength, (**B**) dynamic stiffness, (**C**) phase shift, and (**D**) failure load at different time points. The box plots give the median and the 25th and 75th percentiles. The whiskers give the 10th and 90th percentiles, and the round symbols indicate outliers if present. The dark gray lines and the asterisks (*) indicate differences at *p* < 0.05 identified in ANOVA or ANOVA on ranks and pairwise multiple comparison procedures, such as the Newman–Keuls or Dunn’s method. Number of 0.5% and 5% agarose architecture 1 and 3 model systems: *n* = 6 each, number of 0.5% and 5% agarose architecture 2 model systems: *n* = 7, number of 0.5% and 5% agarose homogeneous controls: *n* = 5, number of 0.5% and 5% agarose open controls: *n* = 6, number of 0.5% agarose material samples: *n* = 8, number of 5% agarose material samples: *n* = 6, number of 0.5% agarose and gelatin architecture 1 model systems: *n* = 5, number of 0.5% agarose and gelatin architecture 2 and 3 model systems: *n* = 6 each, number of 0.5% agarose and gelatin homogeneous and open controls: *n* = 6 each, number of 0.5% agarose material samples: *n* = 8, number of gelatin material samples: *n* = 7.

The mechanical characterization of caffeic acid crosslinked gelatin over time at the two chosen temperatures is presented in Figure 6A–D. Note that the mechanical characterization at each time point was destructive because failure load was measured. Thus, each time point represented a different set of samples and not repeated measurements of the same samples. No differences in the compressive strength (Figure 6A), dynamic stiffness (Figure 6B), phase shift (Figure 6C), or load at failure (Figure 6D) were noted over time, and this was true for incubation at room temperature and 37 °C. However, there was a difference at each time point for compressive strength, dynamic stiffness, and failure load between room temperature and 37 °C (*p* < 0.001), indicating that gelatin mechanical properties deteriorated with incubation at 37 °C, but not over time. The results are illustrated in Figure 6A,B,D. The phase shift at room temperature remained comparable over time. In contrast, it was increased at day 14 at 37 °C (*p* < 0.05), compared to room temperature, indicating that gelatin at 37 °C had become more fluid-like and, thus, more viscoelastic.

### 3.6. Weight Loss of Gelatin over Time at Room Temperature vs. 37 °C

At room temperature, gelatin did not exhibit a measurable weight loss, comparing days 1 and 14. At 37 °C, the weight of gelatin was 67.83 ± 8.49 mg at day 1, which was reduced at day 14 to 21.02 ± 4.81 mg (*p* < 0.05) (results not shown).

### 3.7. Cytotoxicity Tests of Gelatin Crosslinked by Caffeic Acid

**Figure 7:**
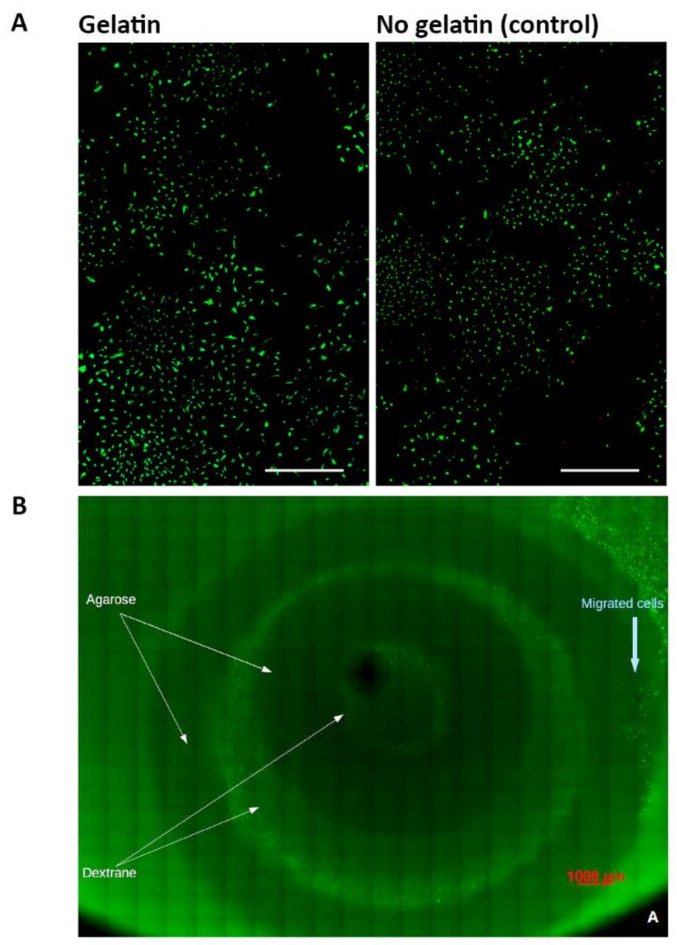
**Representative fluorescence images taken during the cytotoxicity test of gelatin crosslinked with caffeic acid and of architecture 1 fabricated from agarose and a dextran-based hydrogel seeded with human chondrons and mechanically loaded**. (**A**) presents images of human chondrocytes that were incubated with or without gelatin contact to assess cell survival on day 11, using live-dead imaging. Scale bar: 1000 µm. (**B**) presents a fluorescence image of human articular chondrons within architecture 1 that was fabricated from 5% agarose used as a harder biomaterial and from a dextran-based hydrogel seeded with human chondrons used as soft, cell-loaded biomaterial. The image was taken on day 8 after the model system was mechanically loaded on day 7 with a total strain ranging from 0 to 10%. Number of wells with and without gelatin contact: *n* = 6 each, number of mechanically loaded architectures: *n* = 3, number of control architectures: *n* = 2.

Biocompatibility of caffeic acid as a crosslinker for gelatin was tested by calculating the survival rate S. In wells with gelatin contact, S was 86.7 ± 7.7%. S of the negative controls without gelatin contact was comparable with S = 92.4%. The cell density in gelatin-containing wells was 31.5 ± 14.5 cells × mm^−2^ and not significantly different from the cell density of the negative control, which was 49.3 cells × mm^−2^. Notably, in both groups, the cell density was much higher than the seeding density of 5.2 cells × mm^−2^, indicating that the cells had proliferated. Representative images of chondrocytes cultured in a well with gelatin contact and without gelatin contact as control are given in Figure 7A.

### 3.8. Architecture 1 Fabricated from Agarose and a Dextran-Based Hydrogel Seeded with Human Chondrons

Architecture 1 was fabricated from 5% agarose used as a harder biomaterial and from a dextran-based hydrogel seeded with human chondrons used as a soft, cell-loaded biomaterial. Three constructs were mechanically loaded on day 7 using a pre-displacement of 5% followed by a sinusoidal displacement with an amplitude of 5% and a frequency of 1 Hz for, generating a total strain ranging from 0 to 10%, which represent strains that occur from flexion to extension of the spine in the IVD [47], and two constructs remained unloaded and served as controls. All mechanically loaded and non-loaded control constructs maintained structural integrity. There was no significant difference in the survival rate S between loaded constructs (99.9 ± 0.07%) and non-loaded constructs (99.5 ± 0.37%) determined at day 8, indicating that mechanical loading of the model system had no effects on cell survival. Interestingly, on day 8, human chondrocytes from the chondrons had migrated into the 5% agarose rings in both loaded and non-loaded constructs. A representative image of the mechanically loaded architecture 1 and the contained chondrons is given in Figure 7B.

## 4. Discussion

### 4.1. Architecture-Promoted Biomechanical Performance-Tuning of Tissue-Engineered Constructs

The present study designed, fabricated, and tested composite systems of circular components intended to broadly reflect the NP and AF that consisted of biomaterials with different mechanical stiffnesses. This was undertaken to allow studying how slight architectural variations affect the overall biomechanical performance of the composite systems while keeping the material composition across designs comparable. The hypothesis was that such architectural design variations allow reaching higher model system failure loads than those of the individual materials tested either separately or homogeneously mixed.

This study demonstrated convincingly that architectural design variations led to a modulation of the resulting model system failure loads and, more important, that a clever architectural design allowed achieving a TE construct failure load that outperformed the failure loads of the individual materials by far. In the two biomaterial examples used in this study, the model system failure loads were 32.21- and 84.11-fold higher than those of the agarose materials and 55.03- and 2.14-fold higher than those of the agarose and gelatin materials used for system fabrication. This indicates that the mechanical bulk behavior of the model system of circular components under load mimicking the mechanical interplay of the NP and the AF tissues appears functionally important and can be controlled through simple architectural design variations. Those could in the future additionally include angle-ply materials for mimicking AF lamellae, which received much attention in TE, or other sophisticated biomaterials. For example, disc-like angle ply structures (DAPS) made from an electrospun nanofibrous AF and an NP hydrogel core have already been produced [49] and tested in caudal rat spines [50,51], whereas a recent study fabricated cell-seeded angle-ply, multi-lamellated polycarbonate urethane scaffolds [52]. Second, we hypothesized that the here investigated architectural design variations allow modulating other mechanical properties of the model systems than failure load. This was confirmed by this study, but, interestingly, the here presented data collectively highlighted that the compressive strength, dynamic stiffness, and viscoelasticity of the model systems were always in the range of the individual materials used for fabrication. In contrast, the single most important parameter, in which the model systems vastly exceeded those of the individual materials, was failure load. This has important implications for the TE of IVDs and, more generally, for the engineering of load-bearing tissues because TE implants need to withstand the repetitively occurring in situ loads without structural damage. The present study demonstrated that to reach this goal to better withstand high in situ loads, specifically the load-withstanding capacity of current biomaterials can meaningfully be increased by an architectural design that uses multiple circular lamellar components. According to the properties of the materials used for fabrication and the intended application, the ring geometries and amount and order of rings can be optimized. This is a significant advantage of this approach, as it can be used in conjunction with current or to be developed biomaterial for biological IVD therapy. Thus, the contribution of this study is not that we sought to develop another complex IVD construct, but that it introduced an architectural design concept, namely, to optimize the mechanical interplay between the NP and the AF through architectural design optimization of a load-sharing two-component model system of multiple circular, nested, concentric elements. As demonstrated, this optimization allows exceeding the failure load and, thus, the load-bearing capacity of the individual materials used for fabrication.

### 4.2. Material-Independent and Architecture-Dependent Modulation of the Mechanical Properties of TE Constructs

The difference between the architecture designs was that architectures 1 and 2 had both two 4 mm wide hard biomaterial rings. In contrast, architecture 3 had only one 4 mm wide hard ring and a hard center compartment. To account for these differences, the soft biomaterial geometry was varied (Figure 1A,B). These architectural design variations allowed keeping the percentage of the stiff biomaterial across architectures almost constant (63.3% in architecture 1, 56.1% in architecture 2, and 58.13% in architecture 3). Thus, because the composition of all architectures and controls was comparable, the differences in mechanical properties can be explained by the structure, revealing a material-independent and architecture-dependent modulation of the mechanical properties of the model systems. Statistically, this led to architecture-dependent changes in failure load, compressive strength, and dynamic stiffness but not in viscoelasticity. Independently of that, we then defined the “best-performing” architecture as the architecture with the highest average value in either failure load, compressive strength, dynamic stiffness or phase shift, without solely using statistical significance as a criterium for interpretation, following [53]. Interestingly, it was not always the same architecture that best-performed, a term we defined as architecture with the highest average value: agarose architecture 3 best-performed in failure load, whereas architecture 2 best-performed in compressive strength and architecture 1 in dynamic stiffness. For the gelatin architectures, other designs best-performed in each category. These findings might be explained to some extent by the lamellar structure of the architectures. In lamellar composites, the mechanical properties improve with decreasing lamellar thickness [54]. Equivalent findings were described by Chik et al., who indicated that the stiffness and maximal torque of their construct increased with the number of AF-like lamellae [55]. In the present study, agarose architectures 1 and 2 had two 4 mm wide hard biomaterial rings. Their failure load, compressive strength, and dynamic stiffness were statistically somewhat comparable. However, architecture 3 with only one 4 mm wide hard ring was the worst-performing architecture in terms of compressive strength and dynamic stiffness. Interestingly, the best-performing architecture in terms of failure load. Thus, although some mechanical parameter findings were following lamellar composite theory, it is worth noting that the lamellar structure varied here also contained two biomaterials with different properties, 0.5% and 5% agarose vs. 0.5% agarose and gelatin. Only these concomitant variations in design and material properties can together explain the here generated bulk mechanical property variations of the architectural model systems. This appears to be an advantage in musculoskeletal TE because, e.g., the NP is isotropic. Its elastic modulus ranges between 0.05 to 0.1 MPa, while the AF comprises values from 0.1 to 1 MPa [5]. Furthermore, in humans, the elastic modulus of AF lamellae changes from 59 MPa to 136 MPa from the inner to outer region of AF [56]. However, healthy IVDs comprise Young’s modulus from 5 to 20 MPa that decreases with degeneration [47]. The NP pressure can reach in vivo values between 460 and 1330 kPa, depending on the body’s position [57]. Thus, to accommodate the complex design criteria of human load-bearing tissues, such as the IVD, variations in lamellar width, number, and order, combined with choosing specific biomaterial properties, will allow customizing the bulk properties of future load-bearing constructs to a large extent and in a differential fashion.

### 4.3. The Effect of the AF-Like Lamellar Structure on the Mechanical Performance of TE IVD Constructs

As discussed, the elastic modulus of human AF lamellae ranges from the inner to the outer AF region from 59 MPa to 136 MPa [56]. In the outer AF, the linear strain of the inter-lamella is three times higher than that of the intra-lamella (Mengoni, 2015; Vergari et al. 2017), which is reflected by specialized properties, as the human lumbar spine encompasses an AF structure of up to 20 concentric lamellae with a varying thickness from 0.34 and 0.70 mm [58,59,60] and a tensile modulus of the outer AF that is much higher than that of the inner AF [61,62]. Specifically, this lamellar architecture contains the NP under mechanical load [63]. Keeping these anatomical structures in mind, we incorporated three features into our design: (i) a circular, lamellar structure, (ii) an outermost ring that was fabricated from a stiffer biomaterial to prevent soft biomaterial efflux, and (iii) a two-component load-sharing system. With this design, all architectures from both biomaterial sets, 0.5% and 5% agarose vs. 0.5% agarose and gelatin, exhibited a higher failure load than the individual materials used for fabrication. This confirmed the advantage of using a circular, lamellar structure. This architectural design allowed achieving a TE construct failure load that outperformed the failure loads of the individual materials by far. The open controls were designed so that the outward efflux of the soft hydrogel was not restricted by an outer ring made from a harder biomaterial. One reason to build this open control was comparing the individual architectures vs. the open controls allowed dissecting the effects of the presence vs. absence of a biomaterial-confining outer ring using the same biomaterials. Surprisingly, efflux of the soft hydrogel was neither observed in the agarose nor in the agarose gelatin open controls, indicating that material efflux and, thus, architecture-mediated confinement through an outer ring were not significant factors in the here examined model systems. This may be related to high interfacial strength between 0.5% and 5% agarose as well as between 0.5% agarose and gelatin, which was deduced from the absence of outflow of the soft hydrogel. For the agarose sets, high interfacial strength may be based on forming bonds between the stiffer and the softer rings while filling in the hot 0.5% agarose solution. During this process, the contact areas of the two agaroses may have fused due to the high temperature. Gelatin and soft agarose may also build interphase at the contact area of agarose and gelatin because gelatin and agarose are mixable, and this fact was utilized in forming a homogeneous control. Because material efflux was not a significant factor in the function of architecture-mediated material confinement through an outer ring, we further compared the mechanical characteristic between the open controls, between architecture 1 made from agarose vs. agarose gelatin, and between the homogeneous controls. These comparisons suggested that the effects of architectural design were more pronounced than the effects of the choice of materials on failure behavior but did confirm a failure behavior-modulating effect of the choice of material, as all other factors were kept constant in these comparisons. Thus, the presented data demonstrate clearly that the tested architectural designs combined with choosing specific biomaterial properties will allow customizing the resulting bulk properties of future load-bearing constructs in a complex, differential fashion.

Having shown that the effects of architectural design were more pronounced than the effects of the choice of materials, the next question was whether a confining outer ring is necessary for a potential final design. In the human body, the compressive forces exerted on the IVD are converted into tensile stress on the collagen fibers in the AF through the swelling of the NP [2,57,64]. Here, the compressive load was exerted on circular lamellar constructs consisting of softer and stiffer rings. Naturally, the soft biomaterial did not bear as much load as the stiff biomaterial and, thus, must have deformed to a larger extent, converting the compressive axial load in part into radial displacement and tensile load on the stiffer rings. In support of this assumption, the model systems bulged a little at the sides. However, this did not equally apply to both sets of materials, as the failure loads of architecture 1 and the open control were comparable for the agarose model systems but different for the agarose gelatin model systems (*p* < 0.001), indicating that in the absence of an outer ring structure the interfacial strength between 0.5% and 5% agarose must have been stronger than between 0.5% agarose and gelatin. Overall, these comparisons between the agarose and agarose gelatin model systems advocate the presence of an outer ring in the architectural design to allow choosing biomaterials with low interfacial strength.

### 4.4. AF Fiber Re-Enforcement

The mechanical properties of the architecture rings could be further improved by reinforcing the stiffer rings through fiber re-enforcement [57,64,65] and mimicking a multiply architecture as seen in the native AF [5]. Focusing on incorporating fibers into a given AF design is promising because several studies demonstrated that the mechanical properties are highly dependent on AF fiber orientation [36,56,66,67]. Lower fiber angles increase the AF’s circumferential modulus and compressive motion segment stiffness, decrease AF bulging and vertical displacement under compression, and increases rotation under torque [10,40,57,66]. In the present work, the phase shift of the materials used for model system fabrication was not altered by incorporating specific designs, which indicates that the viscoelastic properties of the fabrication materials were maintained. Another factor to consider is interfacial strength. High interfacial strength could theoretically be used to avoid bottoms and lids in model systems or implants. Moreover, after potential implantation, the tasks of the bottoms and lids would presumably be carried out by the vertebrae [1]. Moreover, architectural designs without bottoms and lids would potentially facilitate ingrowth into patient tissues, but one would have to evaluate if newly synthesized ECM molecules might be out-washed with an open architecture [31], which would necessitate construct closure [2,55]. However, construct closure would likely prevent the nutritional supply of the IVD through diffusion and convection between vertebra bottoms and lids and the IVD and, thus, lead to degeneration of the tissue in the long term [68]. In the present study, no efflux of the soft hydrogel during compression was observed. Thus, the bottoms and lids were not necessary for the cultivation and stimulation period and were not included in the final test of the cell-seeded construct.

### 4.5. Biocompatibility of TE IVD Constructs

For experiments that included the dynamic loading of a human chondron-seeded model system, architecture 1 was chosen with a dextran-based hydrogel [46,69] as soft biomaterial and 5% agarose as stiff biomaterial as gelatin degraded at 37 °C and was not suitable for these experiments. In this context, as far as we are aware, this is the first investigation of caffeic acid as a crosslinker for gelatin in musculoskeletal TE research. In 2019, caffeic acid was introduced as a gelatin-crosslinker in bioactive glass scaffolds [42], and earlier work studied caffeic acid in electrospun crosslinked gelatin nanofibers [70]. Beyond the applicability in TE approaches, multiple studies indicated antioxidant, anti-inflammatory, and anticarcinogenic activity of caffeic acid [71]. In the present study, degradation of caffeic acid-crosslinked gelatin was negligible over the course of 14 days at room temperature. At 37 °C, however, gelatin degradation was pronounced, and the model systems lost a large portion of their weight for 14 days incubation. Interestingly, the compressive strength, the dynamic stiffness, the phase shift, and the failure load did not change during this time, but all parameters were much lower at 37 °C than at room temperature, indicating that caffeic acid was not suitable as a gelatin crosslinker, as used here, despite its biocompatibility as demonstrated in the reported cytotoxicity test. Thus, for the dynamic loading of a human chondron-seeded model system, a dextran-based hydrogel and 5% agarose were chosen for fabricating architecture 1. We observed cell migration from the dextran into the cell-free agarose rings in both the loaded and non-loaded scaffolds. Thus, cells did survive in 5% agarose, although the literature suggests cell survival in agarose of concentrations up to 3% [72]. One explanation could be that the cells migrated shortly before the micrographs were taken, and further studies on cell survival during longer time periods would be helpful. Interestingly, the interfacial strength between dextran and agarose was high enough to keep the dextran within the agarose rings during the compression cycle. A dynamic compression in the physiological range of 0 to 10% [47] was applied, and the cells survived in the loaded and in the non-loaded model system equally, demonstrating that the here introduced model system is principally suitable for TE and dynamic loading.

### 4.6. Strengths and Limitations

The present study was associated with limitations, such as the manual fabrication process of the model systems and controls, which likely accounted for the measured variance. Also, the concentricity of the cylindrical rings was visually achieved, which may have led to an anisotropic behavior due to differences in the assembly. Moreover, the scaffolds were not always plane-parallel due to the impreciseness in the manual fabrication process, although the mechanical displacement and load measurements assumed plane-parallelism. We judged that these factors did not lead to large errors because the mechanical characterization was successfully carried out and uncovered substantial differences between architectural designs and materials. For mechanical characterization, the apparent compressive strength was calculated, whereas typically, the equilibrium modulus is measured [17,57]. The choice to measure the apparent compressive strength instead of the equilibrium modulus was made due to the loading situation in the IVD, as high forces applied to the IVD occur as fast impacts during, e.g., walking, running or lifting weight [73], for which the apparent compressive strength is better suited. Of course, in the IVD, other forces are applied slowly or constantly, e.g., when sitting or resting. Arguably the equilibrium modulus might here be more suited, as such activities would lead to an equilibrium reaction of the disc [57]. Furthermore, a specific testing paradigm for AF repair biomaterials has been suggested, which ranges from rapid screening tests to more advanced and also in situ validation tests [3]. These tests have not been carried out in this study because the aim was not to develop a complex IVD construct for potential implantation but, instead, to test the effects of architectural design variations on mechanical properties.

## 5. Conclusions

Relevant architecture-promoted biomechanical performance-tuning of tissue-engineered constructs for biological intervertebral disc replacement can be realized by slight modifications in the design of constructs while preserving the materials’ compositions. In the two biomaterial examples used in this study, the model system failure loads were 32.21- and 84.11-fold higher than those of the agarose materials and 55.03- and 2.14-fold higher than those of the agarose and gelatin materials used for system fabrication. Biomechanical characterization revealed that the single most important parameter, in which the model systems vastly exceeded those of the individual materials, was failure load. The compressive strength, dynamic stiffness, and viscoelasticity of the model systems were always in the range of the individual materials. Minimal variations in the architectural design can be used to precisely control structure–function relations for IVD constructs rather than choosing different materials. These fundamental findings have important implications for efficient tissue-engineering of IVDs and other load-bearing tissues, as potential implants need to withstand high in situ loads. However, concomitant variations in the architectural design, such as variations in lamellar width, number, and order, combined with choosing specific biomaterial properties, allowed customizing the resulting bulk properties to a large extent and in a differential fashion. Thus, the contribution of this study to introduce and test an architectural design concept that potentiates failure load has important implications for the load-bearing capacity of TE implants that need to withstand high in situ loads.

## Data Availability

Datasets are available on request. The raw data and all related documents supporting the conclusions of this manuscript will be made available by the authors, without undue reservation, to any qualified researcher.

## Supplementary Materials

The following are available online at https://www.mdpi.com/article/10.3390/ma14102692/s1, Supplementary Table S1: Composition of the 3D-life hydrogel, Supplementary Table S2: Reagents for the 3D-life hydrogel, Supplementary Table S3: Composition of the chondrocyte cultivation medium, Supplementary Table S4: Composition of the digestion solution for chondron isolation, Supplementary Table S5: Composition of the digestion solution for chondrocyte isolation. Supplementary Figure S1: Experimental Setup.
