# Supplementary material for: Architecture-Promoted Biomechanical Performance-Tuning of Tissue-Engineered Constructs for Biological Intervertebral Disc Replacement
Source: Materials (Basel). 2021 May 20;14(10):2692. doi: 10.3390/ma14102692 (PMC8160686; doi:10.3390/ma14102692)
Supplement: Supplementary file 1 [file materials-14-02692-s001.zip › materials-1177494-supplementary.pdf]

materials

*Supplementary Materials*

# Architecture-Promoted Biomechanical Performance-Tuning of Tissue-Engineered Constructs for Biological Intervertebral Disc Replacement

**Gernot Lang [1], Katja Obri [2], Babak Saravi [1,3], Aldo R. Boccaccini [2], Anton Früh [4], Michael Seidenstücker [4], Bodo Kurz [5], Hagen Schmal [1] and Bernd Rolauffs [1,4,*]**

1 Department of Orthopedics and Trauma Surgery, Medical Center-Albert-Ludwigs-University of Freiburg, Faculty of Medicine, Albert-Ludwigs-University of Freiburg, Hugstetterstrasse 55, 79106 Freiburg, Germany; gernot.michael.lang@uniklinik-freiburg.de (G.L.); babak.saravi@jupiter.uni-freiburg.de (B.S.); hagen.schmal@uniklinik-freiburg.de (H.S.)
2 Institute of Biomaterials, Department of Material Science and Engineering, Friedrich-Alexander University of Erlangen-Nürnberg, Cauerstraße 6, 91058 Erlangen, Germany; katja.glier@web.de (K.O.); aldo.boccaccini@ww.uni-erlangen.de (A.R.B.)
3 AO Research Institute Davos, AO Foundation, Clavadelerstrasse 8, 7270 Davos, Switzerland
4 G.E.R.N. Research Center for Tissue Replacement, Regeneration & Neogenesis, Department of Orthopedics and Trauma Surgery, Medical Center-Albert-Ludwigs-University of Freiburg, Faculty of Medicine, Albert-Ludwigs-University of Freiburg, Engesserstr 4, 79108 Freiburg im Breisgau, Germany; antonfrueh@aol.com (A.F.); michael.seidenstuecker@uniklinik-freiburg.de (M.S.)
5 Department of Anatomy, Christian-Albrechts-University, Otto-Hahn-Platz 8, 24118 Kiel, Germany; bkurz@anat.uni-kiel.de
* Correspondence: berndrolauffs@googlemail.com; Tel.: +49-761-270-26101

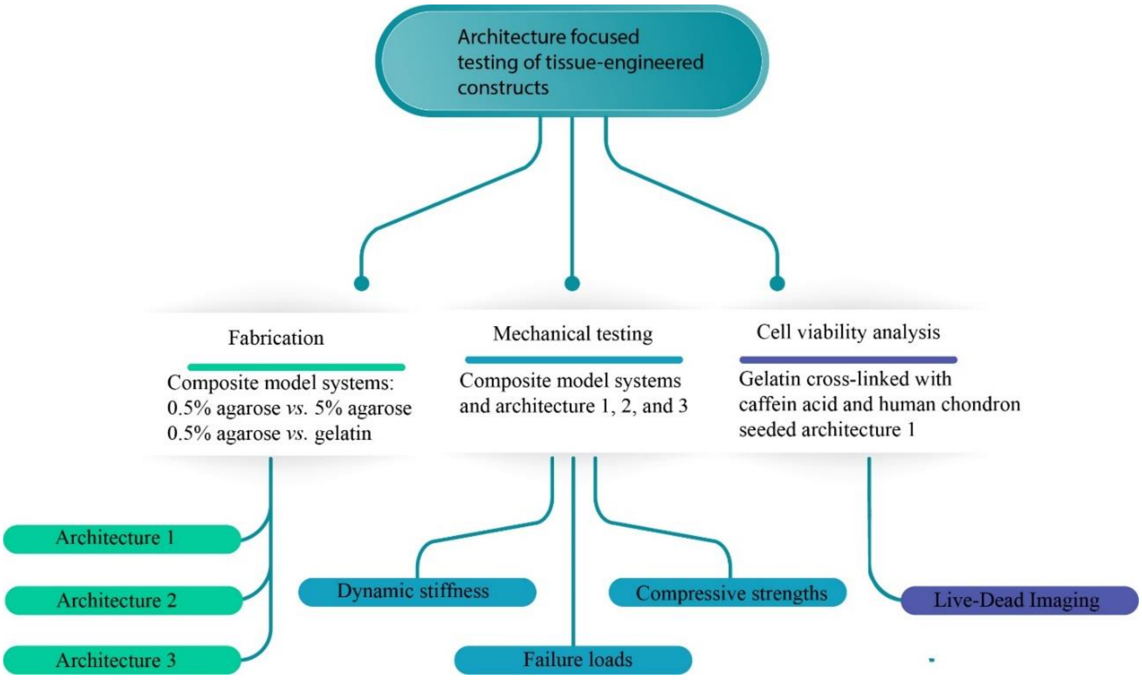


**Figure S1.** Experimental Setup.

**Table S1.** Composition of the 3D-life hydrogel.

| Solution | Concentration | Volume [µL] |
|---|---|---|
| $H_2O$ | | 1.5 |
| buffer solution (pH 5.5) | | 2.5 |
| mal-Dextran | | 5 |
| SH-peptide | | 10 |
| cell suspension | 16x10$^6$ cells mL$^{-1}$ | 5 |
| PEG-Link | | 6 |

**Table S2.** Composition of the reagents for the 3D-life hydrogel.

| Reagent | Composition |
|---|---|
| Mal-Dextran | 30 mmol L$^{-1}$ maleimide groups on dextran<br>5 mmol L$^{-1}$ phosphate buffer |
| PEG-link | 20 mmol L$^{-1}$thiol groups on PEG |
| buffer (pH = 5.5) | 10 g L$^{-1}$ glucose<br>0.5 mol L$^{-1}$ 2-(N-morpholino)ethanesulfonic acid (MES)<br>0.05 mol L$^{-1}$ K Cl<br>1.1 mol L$^{-1}$ NaCl<br>0.2 mol L$^{-1}$ $NaH_2PO_4$ 0.2 g L$^{-1}$ phenol red pH-adjusted with HCl |
| cell suspension | chondrons in PBS (16 × 10$^6$ cells mL$^{-1}$) |
| water | $H_2O$ (cell biology grade) |

**Table S3.** Composition of the chondrocyte cultivation medium.

| Solution | Supplier | Concentration [mg/mL] | Volume [mL] |
|---|---|---|---|
| Ham's F12 Nutrient Mix GlutaMax™ | Life technologies | | 250 |
| DMEM high glucose Gluta-Max™ | Life technologies | | 250 |
| Fetal Calf Serum (FCS) | BioChrom | | 50 |
| Penicillin/Streptomycin | SigmaAldrich | | 10 |
| Fungizone antimycotic | Life technologies | | 6 |
| L-Ascorbic acid 2-phosphate sesquimagnesium salt hydrate | SigmaAldrich | 25 | 0.5 |

**Table S4.** Composition of the digestion solution for chondron isolation.

| Solution | Supplier | Concentration [u/mL] | Volume [mL] |
| --- | --- | --- | --- |
| Dispase 2 | Roche | 2.4 | 8 |
| Collagenase P | Roche | 1.5 | 2 |

**Table S5.** Composition of the digestion solution for chondrocyte isolation.

| Solution | Supplier | Concentration [u/mL] | Volume [mL] |
| --- | --- | --- | --- |
| Dispase 2 | Roche | 2.4 | 8 |
| Collagenase XI | SigmaAldrich | 1500 | 2 |
| Chondrocyte medium | | | 8 |
